# (De)constructing Refugee Vulnerability: Overcoming Institutional Barriers to Ethnographic Research With Refugee Communities

**DOI:** 10.1177/08912416211031645

**Published:** 2021-07-20

**Authors:** Bronwyn Bragg

**Affiliations:** 1 Centre for Refugee Studies, York University, Toronto, ON, Canada

**Keywords:** refugees, research ethics, vulnerability, ethnography

## Abstract

Drawn from 18-months of ethnographic research with resettled refugees living in a mini-enclave in one Canadian city, this article explores what ethnography offers research with resettled refugees. By interrogating the process of securing ethics approval from the Research Ethics Board (REB), I examine the figure of the refugee at the heart of liberal projects aimed at “saving” refugees. I demonstrate that the REB’s reluctance to approve this project stemmed not only from conventional bureaucratic overreach related to ethnographic research but also from an unexamined and problematic idea of what it means to be a refugee. I discuss the gaps between institutionally perceived forms of vulnerability and the actual vulnerabilities that shape life for refugee women. I argue that vulnerability and risk must be understood as contextual and contingent, rather than inherent. Second, I explore the implications of positioning refugees as always already vulnerable on research practice and the value that ethnography offers for overcoming these blind spots.

## Introduction

Recent interventions by critical scholars suggest that research with refugees needs to move away from a “hyper-focus on suffering,” toward accounts that center “the refugees’ rich and complicated lived worlds, the ways in which they labour to have resilient, productive and even heroic lives in displacement” (Lê Espiritu 2014, 9; see also Tang 2015; Ramsay 2017; Lê Espiritu and Duong 2018). These interventions point to the need for methodological approaches to refugee life that attend to the everyday homemaking and placemaking practices of those designated as “refugees.” In this sense, ethnography, a method focused on developing “exceedingly extended acquaintances with extremely small matters” (Geertz 1973, 21) offers an opportunity to explore refugee life in ways that disrupt normative accounts of refugee suffering. Put another way: ethnographic tools allow for accounts of refugee life that shift from universal understandings of “the refugee” to particular, specific accounts of what it means to live under this designation.

And yet, undertaking research on the everyday lives of displaced people is not without its challenges—challenges that, in many ways, exemplify and reproduce problematic ideas about refugees and refugee life. Specifically, and as I argue in this article, the very ideas that we might aim to disrupt about refugee life—that refugees are perpetual victims lacking agency—are the very ideas that hinder access to refugee communities. I make this argument through a careful accounting of my own journey to conduct ethnographic research with resettled Syrian refugees living in Canada. In particular, I explore the bureaucratic and institutional hurdles that sought to limit this research in the form of my University’s Research Ethics Board (REB).

By interrogating the process of securing ethics approval from the REB, I examine the figure of the refugee at the heart of liberal projects aimed at “saving” refugees, and specifically, refugee women and children. I focus here on the figure of the refugee as a means of exploring how refugees specifically are constructed through liberal discourses aimed at “protecting” them and the ways these efforts are reproduced through institutional systems such as the REB. I demonstrate that the REB’s reluctance to approve this project stemmed not only from conventional bureaucratic overreach related to ethnographic research (Haggerty 2004; Newmahr and Hannem 2018; van den Hoonaard 2018) but also from an unexamined and problematic idea of what it means to be a refugee (Malkki 1996; Lê Espiritu 2014). This “idea of the refugee” relies on tropes of refugee life that obscure the ways refugees are political and agentic, with skills and resources to make decisions over if and how they participate in research.^1^

In offering a critique of the REB, I am not suggesting that research with refugees or displaced people is without ethical concerns (Clark-Kazak 2017). As with all research involving humans, there are significant ethical considerations that the researcher must navigate. My argument, however, is that while ethical considerations and responsibilities exist, the *real-life* risks and vulnerabilities facing the refugee families that I encountered were far removed from those suggested by the institutional bureaucracy of the REB. As I argue below, this largely stems from the REB’s problematic understanding of what it means to be “vulnerable” and, in turn, the “risks” of research with populations imagined as always already vulnerable.

I make this argument by documenting the grave concerns the REB expressed related to my project: an 18-month ethnographic engagement with a group of resettled Syrian refugee families living in a series of attached townhomes in a mid-sized city in Canada. These townhomes came to be known as “Little Syria” by residents, service providers, and the local media (cf. Lambert 2017).

My research took place primarily with Syrian mothers and their children, in their homes and in the public spaces in the neighborhood where they spent time. The focus of my work was the placemaking and homemaking strategies of refugee mothers. Building on the interventions by critical refugee scholars (Tang 2015; Ramsay 2017; Lê Espiritu and Duong 2018), my work sought to explore the way specific hyperlocal geographies shape processes of placemaking for women who have been displaced multiple times (Miraftab 2016; Hondagneu-Sotelo 2017).

As a cross-cultural, cross-linguistic, ethnographic project with a group of recently arrived refugees, I was aware that my project involved a degree of risk. I was also deeply aware of my limitations as a researcher, specifically an English-speaking White woman with limited Arabic skills. As such, I spent 6 months prior to applying for ethics developing a methodological approach in tandem with members of the Syrian community where my research took place. I hired a Syrian woman who lived in Little Syria to translate and support outreach with her neighbors. Together we developed a research protocol that she and others in the community felt ensured the safety of potential participants, while also attending to the cultural and linguistic specificities of the Syrian community in East Calgary (a process described in detail below).

Despite this, the REB remained reluctant to grant ethics approval for my project. They considered resettled Syrian refugees to be “highly vulnerable” and thus, the research “high risk.” They encouraged me to consider partnering with a settlement agency rather than conducting fieldwork in the community, and they expressed profound concerns over my lack of language ability in Arabic, despite the engagement of an Arabic-speaking, Syrian research assistant, and translator. I argue that the concerns expressed by the REB were largely out of touch with the realities and concerns facing women in Little Syria.

The article proceeds as follows: I begin by providing the context for this research, including the policy landscape that surrounded the arrival of Syrian refugees in Canada in 2015–2016. I describe my methodological approach in detail and situate myself in relation to the research. In the next section, I describe navigating the REB at my university and the REB’s objections to this project. In this section, I explore the twofold concerns of the REB, first the general overreach of REB and their concern over “vulnerability” and, second, the REB’s mobilization of particular tropes surrounding refugee life. This second point is elaborated in the final three arguments of the article, where I demonstrate the problematic “idea of the refugee” at the heart of REB’s objections to this research.

## Background & Methods

### Syrian Refugees in Canada

In November 2015, the newly elected Canadian Liberal government launched the “Syrian Refugee Resettlement Initiative” (SRRI) with the goal of resettling 25,000 Syrian refugees before the close of the year. The government took what they have since described as the “whole of society approach” (Kiziltan 2020) which sought to bring together various levels of government and a range of civil society stakeholders around the shared goal of “welcoming Syrian refugees.” The SRRI was unprecedented with respect to the scale and scope of citizen engagement. Thousands of Canadians became involved: raising funds, donating goods, and joining groups to help sponsor a refugee family.^2^

Between 2015 and 2017, 40,000 Syrian refugees arrived in Canada through the SRRI.^3^ Of those, approximately 2000 Syrian refugees arrived in 2015–2016 in Calgary, Alberta, a city of 1.3 million in western Canada, where this research took place (IRCC 2017). The rapid arrival of refugees, coupled with large family sizes and limited financial resources for housing, meant that many Syrian families ended up living in Calgary’s more affordable neighborhoods on the east side of the city. In particular, a significant number of refugee families secured housing in a housing complex in the east Calgary neighborhood of Forest Lawn. This particular housing complex is made up of attached townhomes around a large green space. The housing complex came to be seen—by Syrian families—as a highly desirable place because of the affordability of the properties, the style of home, and the presence of other Syrian families. This area became known to residents and service providers as “Little Syria.” It is difficult to get an accurate number of how many Syrians live in Little Syria at any one time, but at the time of my research, it was estimated that between 35 and 40 Syrian families were living in the complex.

### Methods & Setting

Prior to applying for ethics approval from my university’s REB, I spent approximately 6 months (June 2016 to January 2017) working to get a handle on the dynamics of the newly arrived Syrian community in Calgary. I did this through informal interviews with a range of stakeholders, service providers, volunteers, and private sponsors. I also connected with a few recently arrived families, including by participating in an informal language exchange with a Syrian woman and her children.

It quickly became evident that a major challenge would be the considerable language barrier between me and potential research participants. Most of the women in Little Syria had very limited English at the time of this study, and I had very limited Arabic. Despite mutual efforts to learn the other language (I was studying Arabic, and the women I met were in English classes), the language barrier remained significant.

Between the language barrier and the fact that researchers cannot simply show up on someone’s doorstep and start asking questions, I decided to hire a research assistant who lived in Little Syria to support this work. I reached out to a leader from the Syrian community, who had been in Canada for over 20 years and who had been involved in the Syrian resettlement, to help me find a research assistant. He was encouraging about my research and connected with me with Haya (pseudonym), a mother of four, originally from the city of Homs in Syria, who he believed was well-regarded and trusted in the community. It was with Haya that I developed the research methods and protocol for my fieldwork. She emphasized the concerns over trust in the community and discouraged note-taking or recording conversations. Haya suggested that the best way forward would be to have a series of informal conversations and visits with women in the community, explaining the research and my interests as we met women. Haya believed that women would be happy to meet a Canadian woman of similar age who could answer potential questions about life in Canada.

Haya and I began meeting several times a week and I would accompany her to meet other women in the community. Most of our time together was spent having informal conversations with the Syrian women who were her neighbors. With a few exceptions, there were no men present during these interactions.^4^

As my time in the field progressed, I started to spend more time with two families. One was the family of my community connector, Haya, and the other was a friend of Haya’s named Ameera. Ameera and Haya became key informants, answering my unending questions and helping explain context and details that I inevitably missed.

In addition to participant observation, Haya and I conducted interviews with 13 women living in Little Syria and then conducted follow-up interviews with six of those women. All the interviews with the exception of three took place with the participant speaking in Arabic and Haya translating. Three women felt comfortable enough in English to respond to my questions in English (with occasional translation assistance from Haya). The interviews were exclusively with the women in the home (except for one). All the participants except one were married, with children, and lived with their husbands. One participant was a widow. Every interview was dominated by the presence of children. The average number of children in each household was between four and five. One woman had seven children and one had two at the time of the interview (though she was pregnant with her third).

### Situating the Researcher

Cross-cultural, cross-linguistic research with refugee women is not without risks. Sherene Razack writes: “There is usually a very significant difference between the refugee storyteller and the listener. That difference is one of enormous power. . .Further, power relations in the refugee context come dressed up as compassion” (1996, 171). As Razack correctly points out, these relations are steeped in problematic power dynamics haunted by the humanitarian desire to “help.” Writing about a similar study of Syrian refugee mothers in Ontario, Maghbouleh et al. (2019) write about the challenges of conducting research with Muslim women from Syria: “our project risked reproducing uncritical, homogenizing, and Orientalist ideas about gender and power, particularly in the context of mothers’ forced migration and resettlement” (2019, 487–488).

Certainly, the language and cultural differences presented a real-world challenge to undertaking this research: Despite years of experience working with immigrants to Canada, I had never worked directly with a population that had arrived so recently. In my first encounters with refugee families, the language barrier overwhelmed me. It is no surprise that some of the first words I learned in Arabic were, *“shouaya shouaya”* which means “Slowly! Slowly!”—as in, “Slow down! You’re speaking too quickly!” My concerns about not speaking Arabic were echoed by the BREB that told me that for research with this population, “Arabic seems like an essential skill.”

My concerns over language went beyond the issue of mutual comprehension. For decades, feminist researchers—anthropologists, sociologists, and geographers—have concerned themselves with the questions of power and representation in research (Trinh 1989; Visweswaran 1994; Razack 1996; Hyndman 2000). These concerns are central to research across not only language and culture, but also where there are great imbalances of power, such as research between an English-speaking, Canadian-born researcher, and Arabic-speaking, recently arrived refugees to Canada. Given all the epistemological challenges of research and the very real concerns about representation and power, is research across language and culture barriers, with “vulnerable” groups even worth the effort? Or is it too risky? Too problematic? Too fraught?

While I shared a similar age and gender identity as my research participants, our life experiences differed considerably. This was reflected in a conversation I had with my Syrian research assistant, Haya, a few weeks after meeting, we realized we were born a year apart. I had assumed that she was several years older than me. She had four children, her oldest was 12 and she just *seemed* older. On her side, she found it incredible that I, a 33-year-old married woman, did not have any children. While my life had been a journey of personal and professional development, graduate school, travel, and now I was settled down living near my family in Calgary, the last 10 years of her life had been a tumultuous journey of dislocation, relocation, and struggle. She was now raising a family in a corner of the earth she had never dreamed she would visit, let alone make a life in. Her extended family was scattered across Turkey, France, and Syria. Haya is also a devout Sunni Muslim. She assumed, as happened a lot, that I was Christian. Over time she came to realize that I was actually not religious at all, which was perhaps more strange than if I had been Christian. Thus, we both struggled to locate each other in relation to one another.

My position as a researcher, especially as an English-speaking, White woman of a certain age, meant that I followed in the footsteps of journalists, armies of volunteers, sponsors, and other helpers who intersected with Syrian families in their resettlement to Canada. It was in this way that I was received by the families I interviewed—as both another curious Canadian and as a potential helper. Could I make a quick phone call for them? Could I read this document from the school? The teacher wants to move my son to regular class—should he move or stay in LEAD?^5^ How much does university cost in Canada? The pharmacist gave me this long thing to read along with my prescription, what does it say? Are there drugs at the schools? Why is the waitlist for Calgary Housing so long? My LINC^6^ teacher says I have to move to level 5, but I do not feel ready, why cannot I re-do level 4?

Ultimately, it was through these encounters that I found my footing as an outsider and researcher in Little Syria. While Syrian refugees in Canada were overwhelmed with attention and enthusiasm when they first arrived, by the time I entered the field (1.5 years after they arrived in Canada), there were few Canadian volunteers and sponsors still involved. This meant there were few opportunities for the women of Little Syria to talk to or engage with “a real Canadian” (a description of me offered by several participants). My presence in their homes offered an opportunity to ask questions that they may not have had the opportunity to ask others. Much of my time in Little Syria was spent working with families navigate the impenetrable systems that govern life in Canada: Registering children for school bus pick-up, making an online appointment to have blood drawn at the lab, filling out long and complicated forms from the Canada Revenue Agency, helping mothers apply to get new babies their birth certificates and on and on.

In this way, I never became an “insider” in the community, but perhaps something closer to what Sandra Bucerius (2013) described as a “trusted outsider.” This position did not resolve the complexity of representation and difference that attends research of this nature, but it did allow me to navigate these challenges.

## Navigating Institutional Barriers

My methodological approach was informed by two general principles grounded in both my lived experience (working with refugees) and in decades of academic research centering the agency of refugees at the core of this work (Malkki 1996; Tang 2015; Besteman 2016). My experience working with the Syrian population specifically, and migrant/refugee/immigrant communities more generally, confirmed what the academic research described: That refugees are skillful at accessing the supports and resources needed for their settlement journey—this despite the seemingly overwhelming structural barriers facing resettled refugees in Canada.

I was aware that my research project involved a degree of risk due to the nature of the research I was proposing. This included language differences, literacy barriers making long written consent and recruitment forms problematic, and possible mental health issues stemming from trauma. I also wanted to conduct research in the homes of refugees, and not in settlement offices or at a partner agency, which brought other risk factors into play.

In my ethics application, I explained how I planned to mitigate possible risk: I had hired Haya, an Arabic-speaking, Syrian woman from the community, to accompany me for all my interactions with refugee families, including the consent process and interview. I explained that the goals of my research were to build an understanding of the settlement experiences of refugee families (i.e., their lives in Canada), and not rehashing their experiences in Syria during the war or their flight from Syria.^7^ I cited literature and research around using verbal consent instead of written consent to avoid embarrassment and concerns over literacy. In sum, I felt that I had understood and described the possible risks associated with my research and had addressed those concerns as well as I could.

In Canada, university REBs are guided by the Tri-Council Policy Statement on Ethical Conduct for Research Involving Humans (TCPS2). This lengthy document spells out the ethical guidelines for research involving human subjects, including as they relate to risk and research with “vulnerable” populations. The TCPS2 states that for research to be considered high risk, “the probability and magnitude of possible harms implied by participation in the research” must be “greater than those encountered by participants in those aspects of their everyday life that relate to the research” (TCPS2 2.8). What was evident to me, was that my research, while focused on a purportedly vulnerable population, did not imply a greater risk than “those encountered by participants in those aspects of their everyday life that relate to the research.”

Despite my convictions, the REB at my university disagreed with my assessment that my research was “low risk.” The Board wrote:

It appears that the population sampled (i.e. Syrian refugees) is *highly vulnerable and this study involves high risk*. Please reconsider your response and provide more detail on the risks and vulnerabilities of each group of participants involved in this study (emphasis added).

The decision to designate resettled Syrian refugee families as “highly vulnerable” and a “high risk” research proposition reflects a twofold challenge for ethnographers interested in studying refugees. The first challenge relates to the bureaucratic hurdles that need to be overcome to research with refugee migrants *without* the conventional gatekeepers, such as immigrant-serving agencies, that are preferred by Ethics Boards for this kind of research. This a challenge for ethnographers *in general* who engage in research that Ethics Boards find inherently difficult to manage.

The second challenge relates to the stickiness of the term “vulnerability” and who, precisely, is vulnerable. As I argue below, in designating resettled Syrian refugee families as highly vulnerable and a high-risk research proposition, the REB reinforced problematic ideas about refugees as inherently vulnerable, lacking agency, and therefore a “high risk” research proposition. In so doing, the Board employed an uncritical and problematic idea of the refugee that reflects a wider social impulse that requires that refugees are always already in need of saving and protection (Malkki 1996; Ticktin 2016).

### Bureaucratic Hurdles

There is an extensive body of scholarship describing the increasing power of REBs^8^ and the way they curtail and limit certain kinds of research (Haggerty 2004; Chin 2013; van den Hoonaard 2018). Much of the critique stems from the translation of medical ethics by social science review boards: scholars suggest that social science has uncritically adopted much of the language and criteria of medical ethics while failing to account for the distinctions between medical and social science research (van den Hoonaard 2018).

Ethnography in particular faces scrutiny from REBs which seek to understand *in advance* the potential harms and outcomes of research—something that is challenging with the fluid nature of ethnographic research (Haggerty forthcoming; Newmahr and Hammen 2018; Delamont and Atkinson 2019). Ethnographers point to the challenge, for example, in securing informed consent from their participants. While individual participants may sign consent forms, ethnography by definition explores *groups* and interactions within and between group members, it may not be possible and/or realistic to secure consent from all members of the group (Delamont and Atkinson 2018).

This relates to the role of the REB in protecting *institutions* (in this case universities) from the potential “risks” associated with research. Scholars in this area point to the growing bureaucratization of universities and the shift toward administrative oversight of research (Chin 2013; Newmahr and Hammen 2018). Newmahr and Hammen write that for ethnographers, “the growing administrative power of the REB is a death sentence: a slow process, but fatal nonetheless” (2018, 6).

In a critique of REBs, Elizabeth Chin writes:

Fine-grained work with the homeless, battered women, transgender youth, and drug users often face challenges from REBs that, primed as they are to protect institutional interests, find it difficult to imagine how the research can be undertaken in ways that render the institution damage-proof (2013, 190).

To that list of “vulnerable” populations, I would add refugees. As Chin rightly identifies, research with these populations raises red flags for institutions “primed to protect institutional interests.”

A related school of thought points to what Kevin Haggerty has aptly described as “ethics creep” (2004), where REBs have “unintentionally expanded their mandate to include a host of groups and practices that were undoubtedly not anticipated in the original ethics formulation.” (Haggerty 2004, 392). The unknowability of what risks and potential harms *might be* encountered through ethnography leads, as Haggerty notes, to “a considerable degree of speculation into the research ethics review process” (forthcoming). It is likely that those serving on REBs have limited familiarity with ethnography as a research method and/or the community proposed for study, in turn: “research ethics boards can exoticize the research participants and settings. . .and over-estimate the nature of risk involved” (Haggerty forthcoming).

This final point centers on concerns related to the concept of vulnerability and its problematic application by REBs. van den Hoonaard (2018) traces how the term “vulnerability”—which has a specific meaning for medical ethics—was adopted by social science research ethics. The problem for social science, argues van den Hoonaard, is that a term that has specific meanings in medicine becomes problematically attached to various populations in social science research. In medicine, the term refers to the capacity of a research participant to give informed consent. This same standard is applied in social science research, “but now [ethics regimes] see vulnerability as a significant, stand-alone component that researchers in the social sciences must also consider” (2018, 308). The concept of vulnerability is not only “fixed and unmovable” (certain populations/groups *are* or *are not* vulnerable) but also “vague and ill-defined” (van den Hoonaard 2018, 314). This application of the “doctrine of vulnerability” both ignores how research participants might see their own life circumstances, as well as the possible benefits of a research encounter (2018, 319).

### Refugees and the Liberal Will to do Good

What are the implications of describing (Syrian) refugees as “highly vulnerable” and, in turn, research with these refugees to be “high risk?”

My application to the REB was initiated in the winter of 2017, approximately 18 months after the wide circulation of the photo of Alan Kurdi—the 3-year-old Syrian child who drowns with members of his family attempting to reach safety. This image provoked a powerful emotional response on the part of many Canadians (Walton-Roberts, Veronis and Hamilton 2020). This photo and the emotional response it engendered meant that refugee issues became a potent issue in the 2015 Federal election in Canada; and ultimately led to the SRRI, which saw the resettlement of some 40,000 Syrian refugees in the next 2 years.

For many, the lifeless body of a child on a beach came to symbolize the Syrian crisis. For example, in their study of Private Sponsors, Macklin and colleagues found 83% of respondents were “very” or “somewhat” influenced by the death of Alan Kurdi to become involved in private sponsorship (2018, 49). His death provoked a profound response on the part of many Canadians to *do something* to protect Syrian refugees.

Critical studies of refugees and humanitarianism describe the way certain highly gendered and sensationalized images circulate to produce refugees as enduring victims (Hyndman 2010; Ticktin 2016; Lê Espiritu and Duong 2018). These images have the effect of focusing attention on displacement onto the bodies of particularized Third World victims, and away, as Lê Espiritu and Duong write, from “a serious analysis of the geopolitical conditions that produced their displacement in the first place” (2018, 587). In this way, for many Canadians who became involved in the Syrian Resettlement Initiative, their understanding of Syrian displacement was inextricably linked to the tragic image of a dead child. While this produced a powerful emotional (and, in turn, political) response to the suffering of Syrian refugees—it also had significant repercussions on the kind of reception Syrians received in Canada.

Miriam Ticktin describes the way certain images provoke specific humanitarian responses. Humanitarianism, she suggests, relies on a “narrow constellation of emotions” (2016, 256): compassion, empathy, benevolence, and pity. It requires that whose humanitarian responses are aimed at remain innocent: “Innocence establishes a hierarchical relationship: those who care and those who are cared for. Of course, care is welcome: *but what does it mean to be welcomed as a victim, passive, and unable to take care of oneself?*” (2016, 260, emphasis added).

Ticktin’s description of refugees as “victim[s], passive, and unable to take care of oneself” was reflected in the REB’s description of the (resettled) Syrian refugees in Canada—“highly vulnerable.” In designating these refugees as such, the REB ignored the years of struggle and survival that had brought these refugees to Canada. What of the decisions and choices these families made every step of the way in their journey from Syria, to a country of first asylum, and then to Canada? While “victims” of the conflict in Syria and a global refugee system that so regularly fails displaced people, these families were also agentic: they demonstrated considerable skill at navigating complex bureaucracies and precarious living situations to keep their families intact and raise their children. As such, they were far more than simply victims, passive and unable to take care of themselves.

Problematic representations of refugees as perpetual victims are not new. Liisa Malkki’s research from the 1980s on Hutu refugees from Burundi living in Tanzania found that the figure of the refugee is shaped by normative understandings of what a refugee should be—“exemplary victims” (1996, 384). Malkki explores the visual representations that accompany refugees—these images tend to erase the individual, particular, specific histories of people, and instead, they are transformed through images into “anonymous bodies” (1996, 389). Through humanitarian logics and media representations, refugees become what Malkki describes as “speechless emissaries”—“in universalizing particular displaced people into ‘refugees’ - in abstracting their predicaments from specific political, historical, cultural contexts - humanitarian practices tend to silence refugees” (1996, 378).

In identifying resettled Syrian refugee families as “highly vulnerable” and my proposed research as “high risk,” the Ethics Board reinforced the normative assumption that refugees are passive victims, “speechless emissaries” as Malkki described them, who would likely be revictimized through research. This echoes Yen Lê Espiritu’s claim that much scholarship on refugee life often “[ignores] the refugees’ rich and complicated lived worlds, the ways in which they labour to have resilient, productive and even heroic lives in displacement” (2014, 12).

## Constructed Refugee Vulnerability

The REB reflected little interest in situating the research I was proposing within the current social, political, and economic realities of resettled Syrian families. The refugees with whom I was working had been in Canada for 2 years by the time I encountered many of them. And they had been outside of Syria for another three to 4 years prior to that. Their concerns were less about their human security, and more about the business of “settling” in a strange, new country. From the moment they decided to come to Canada, their lives were governed by complex systems of humanitarian governance (Garnier, Jubilut and Sandvik 2018). First, the humanitarian regime in their country of first asylum, then the Canadian refugee resettlement system, which continued for their first year in Canada either in the form of Refugee Assistance Program (RAP) providers or through their Sponsorship Group. As that first year of formal support ended, these families continued to be regulated and managed by the provincial welfare system, the immigrant-serving sector, language classes, volunteers, sponsors, and the “mainstream services” (Canadian schools, medical systems, police, and social workers) that govern and regulate Canadian society.

The concerns of the Ethics Board ignored the profound ways in which the everyday lives of these families are already caught up in English-speaking Canadian-thinking systems—these were not always apparent, visible, or understood by refugee families, and often these systems failed these families. Ethnography provides one small way of documenting the perspectives and experiences of those subject to these systems.

The remainder of this article highlights three examples of what I observed in my early months of fieldwork that confirmed this disconnect between the *perceived* vulnerability attributed to these families by my university, and the everyday, real-life challenges they faced navigating Canadian systems and services.

### Privileging Participation

A central intervention of critical refugee studies is to argue for researchers to pay closer attention to the “improvised, fluid, and alternative, homemaking, healing, and survival strategies” that refugees create (Lê Espiritu and Duong 2018, 588). These strategies are necessarily spatial and often take place in spaces “that exist behind, between, and beyond [public(ized) spaces]” (ibid). Yet *accessing* places “behind, between and beyond” public space is often made challenging by institutional processes that privilege some spaces over others.

This was made clear to me by the REB’s concern over the specific *location* of my study. Why they asked, could I not conduct this research in partnership with a local refugee-serving agency? Perhaps in their offices? It would be, the REB suggested, “less coercive.” Second, should I push forward with research in the private homes of refugee families, the REB expressed concern over how I might deal with incidents of child abuse which I would no doubt encounter in my research. The Board asked: “How will the researcher deal with a situation in which a participant reveals parenting actions that may be considered abusive in Canada?” I address these two concerns in order.

By suggesting that I undertake this research in partnership with a refugee serving agency, and not in a community setting, the Board inadvertently privileged the participation of *certain kinds* of refugees. Specifically, those who access services on a regular basis and can be easily accessed through a settlement agency. The problem, however, is that a significant number of refugees do not access these services, and this is especially true of refugee women, whose multiple family and caregiving responsibilities (both in Canada and in maintaining ties to family members “back home”) often prevent them from accessing formal services.

Research on the use of settlement services in Calgary found that nearly 47% of newcomers had never accessed formal settlement services (Calgary Local Immigration Partnership 2018). For recently arrived Syrian women—most of whom did not drive and who had multiple young children—finding their way to a settlement agency was fraught with challenges. A key area of focus for my research was on the nature of “settlement support” that refugee women access. I knew that if I worked through an agency, I would only see the limited population of those who access services.

My assumptions about the ways in which refugee women in Little Syria interacted with settlement agencies were confirmed after a few months of fieldwork. The vast majority of the women I interacted with were not engaged in formal settlement programs. While they may have attended English classes upon their arrival in Canada, most had stopped going because they had become pregnant and had very young children at home. When asked where they turned for assistance and support, most of the women in Little Syria identified other Syrian families or Arabic-speaking neighbors who would offer assistance. For example, rather than turning to the local service provider for assistance with their tax returns, most families in Little Syria paid a Lebanese-Canadian woman who was the proprietor of a Lebanese shop in the neighborhood, where Syrians bought *halal* food and other cooking staples. Women in the community trusted her far more than the settlement agencies that required making an appointment, traveling downtown, and filling out complex paperwork.

In addition to this practical support, in undertaking research in the community, I was able to observe the relations of care and support between women in Little Syria. In this way, I came to see the fragile and emergent social ties between women that helped buffer the stress and feelings of dislocation that accompanied their resettlement to Canada.

These insights would have only been available to me through ethnographic research in the community—had I attempted to recruit participants through a formal settlement agency, I would have missed the importance of local connections and the accounts of women who are not engaged in these formal services.

In privileging the “public” space of a settlement agency (perceived by the Board as less coercive), the REB was likely also seeking to mitigate the possible risks that attend research in private homes. While research in private homes does involve a degree of risk (for both researchers and participants), these were addressed in my ethics application. Yet the question posed regarding “parenting actions that may be considered abusive in Canada” reflected a very specific set of assumptions by the REB.^9^

First, the assumption that immigrant/refugee families use “abusive” approaches to parenting on such a regular basis that I—an occasional visitor—would witness these practices. And second, that refugee families are unaware of Canadian laws and norms around which “parenting actions” are considered abusive. Both these assumptions were wrong—I never witnessed anything resembling “parenting actions that may be considered abusive in Canada,” and families were *very* clear about Canadian censures on corporeal punishment, as these messages are almost *over-communicated* to refugee families upon arrival in Canada.

This is where the perceived risks to families imagined by the REB and the *real* risks to families diverge. In the REB’s framing, refugee parents (1.5 years after they arrived in Canada) would be woefully unaware of “appropriate parenting actions” such that they would be at risk of disciplining their children inappropriately *in front of a relative stranger*. This assumption was painfully disconnected from the lived reality of refugee families who were the subject of constant and unending surveillance and scrutiny over their parenting practices, and who were deeply aware of Canadian “norms” vis-à-vis parenting.

As I write elsewhere (Bragg 2020), Syrian refugee families in Canada—along with another refugee/immigrant, low-income, and racialized families (Ong 2003; Kershaw 2010; Creese 2011)—endure specific forms of surveillance. These families are expected to comport to an imaginary standard of White middle-class family norms. Refugee mothers work hard to navigate these tacit—and not-so-tacit—expectations shaped by settlement providers, social workers, teachers, volunteers, and private sponsors. The Syrian women I met in this study would regularly repeat back to me the various rules and instructions they received upon arrival in Canada: Children cannot be left alone; Children must be supervised when they play; Children must wear helmets when they ride bicycles; In Canada, children can call the police if they are scared.

In treating refugee mothers as potentially deviant and at risk of abusing their children, the REB reflected a wider institutional framing of refugee mothers and minoritized parents. While this is problematic on its face, it also reflects a great misunderstanding on the part of the REB about the *actual* vulnerabilities facing refugee mothers; including from the systems of governance and surveillance that surround these families. Further, contrary to the belief of the Board, these mothers—out of sheer necessity—had become adept at navigating these systems and were careful to reflect to “outsiders” (such as myself) that they were clearly complying with the many rules imparted to them by various gatekeepers of Canadian society.

### The Messiness of Cross-Cultural Encounters

A central concern of the REB was my lack of proficiency in Arabic. Along with describing fluency in Arabic as an “essential skill,” the REB continued to insist (even *after* granting ethics approval) that “learning the local language or a local lingua franca is expected by anthropologists prior to the start of research or, if language learning materials are not available, during research.”

While I shared the Board’s concerns regarding language, I also maintained that this research could be accomplished by a non-Arabic speaker. I believed this because I was observing on a regular basis the way Syrian families were already caught up in English-speaking systems and had regular engagements with non-Arabic speaking members of Canadian society. Indeed—this cross-cultural, cross-language engagement was the basis of the Canadian government’s “whole of society” approach to the Syrian resettlement which relied on the passionate engagement by a wide spectrum of Canadian society who became involved in the Syrian Resettlement Initiative.

These cross-cultural encounters merit a paper of their own, but suffice to say, they reflected the inherent complexity and messiness of cross-cultural engagement. One site where this complexity was amply visible was the relationship between Private Sponsors and refugee families. The Syrian Resettlement Initiative saw unprecedented levels of interest in private sponsorship by groups of volunteers, many of whom had never participated in refugee sponsorship in the past (Macklin et al. 2018). Through private sponsorship, groups of “regular” Canadians came together to sponsor a refugee family from Syria. Once the family arrived in Canada, the Sponsorship group was responsible for the first year of funding for the family. The degree of engagement between Sponsorship Groups and families varied widely (Agrawal 2018). It was not unusual, however, for close relationships to develop between members of the sponsorship group and the refugee family. For example, I knew of sponsors who had been present at the birth of new Syrian babies, or who interacted daily with Syrian families, months into their arrival. In other cases, the relationship between sponsor groups and refugee families frayed over irreconcilable differences.

In sum, cross-cultural engagements between non-Arabic speaking members of the Canadian public and Arabic-speaking Syrian families were happening from the moment Syrians arrived in Canada. They reflected the full spectrum of what can happen in cross-cultural, cross-linguistic interactions: fragile but potent solidarities across differences, as well as hurt feelings, misunderstandings, and confusion. Indeed, it was precisely the nature of these interactions, and how refugee women felt about them, that I sought to understand through my research. And yet, the REB, failing to understand the complexity of everyday life for these families—caught up as they were in English-speaking, Canadian systems of governance, policy, and interpersonal relations—continued to insist on the impossibility of research undertaken by a non-Arabic speaker.

I outline below an example of the kinds of interactions I was observing and participating in prior to the “formal” commencement of my research:

It was a warm day in July of 2017 and I had been sent to the house of a Syrian family living in a basement apartment not far from my own home. The apartment was on a leafy residential street, it was a fourplex with two apartments up and two apartments in the basement. The family was moving to another apartment in a different neighbourhood. I had not met them before, but I had met one of the women who had helped sponsor them to come to Canada through her church. It was this sponsor [Sheila] who had sent me to visit the family. Sheila had been involved in private refugee sponsorship through her church for several decades and was thus well versed in the system. She had been heavily involved in the daily lives of this family, including attending the birth of the youngest member of the family. Recently, however, there had been a falling out, due to differences of opinion between Sheila and the family about their decision to move. Sheila had secured an apartment she felt was appropriate for the family (budget, location etc.), while the family had found a different apartment they preferred. The apartment preferred by the family meant the children would have to move schools (something Sheila felt would be disruptive) and was in a less desirable neighbourhood (according to Sheila). Yet the family felt that the apartment Sheila had chosen was not appropriate for their needs. The kitchen and living room were one large space, which meant it would be difficult for the family to socialize with guests with women in one room and men in the other (as was preferred by some Syrian families). This led to a conflict and ultimately Sheila’s decision to stop supporting the family. They had, after all, been in Canada for well over a year, and they were clearly ready to make their own decisions (according to Sheila). Despite this, the family had requested assistance with changing their addresses with the various systems that they interacted with (Canada Revenue Agency, Immigration etc.) Sheila asked if I could go assist, as a neutral party. Upon arrival in the family home, I was handed a folder containing the family’s essential paperwork: Financial documents, immigration papers, tax returns, school registration and so on. I spent over two hours in the home, attempting to change their mailing address with various government agencies over the phone. This was a futile exercise as I did not have any of the appropriate authorizations from the Government to act as a proxy for the family (though Sheila did) and Wahid – the father – was not comfortable speaking in English to the various government representatives on the phone. The older children, who spoke English, were also not authorized to speak on behalf of the family. We ultimately decided that change of address would be better accomplished through mail rather than over the phone, so I took the relevant information (here including highly sensitive personal information), returned to my home, wrote a series of letters, returned to Wahid’s apartment, where he and his wife signed the documents, and then mailed them to the Immigration and CRA. (Fieldnote excerpt, 071317)

This interaction between a relatively recently arrived Syrian refugee family and a member of the non-Arabic speaking, non-Syrian Canadian public was not unusual for this period of the resettlement initiative. I was regularly handed documents that contained private and sensitive personal information, in many cases, I often completed this paperwork on behalf of families—translating the document from technocratic government language to plain language as I went. This was also the case with sponsors and volunteers, who assisted with all manner of paperwork as well as other necessities of daily life. At one point I was even inadvertently roped into an impromptu driving lesson with a Syrian woman who was trying to get her more advanced license. Many sponsors and volunteers accompanied Syrian refugees to doctor’s appointments and translated highly sensitive medical information to refugees—despite not speaking fluent Arabic. In highlighting the complexity of these interactions, I am not suggesting that they are absent enormous ethical challenges around trust, privacy, and power relations. What I am suggesting, however, is that ethnographic research has an essential role to play in examining how these relations feel from the inside.

### Implications of Constructed Refugee Vulnerability

Much of the literature on REBs and risk points to the way REBs are more interested in managing risk to the institution rather than risk to participants (see Chin’s quote above). Yet the concerns of the Ethics Board were not inconsequential. Indeed, they reflected broader institutional understandings about refugees, their vulnerability, and their ability to consent to research.

This perception of the heightened vulnerability facing Syrians impacted my own methodological approach in ways I was not even aware of until much later in my research process. For example, I structured my research around the *settlement* experiences of refugee families. I committed to the REB that my questions would focus solely on the experiences of families since their arrival in Canada. I would not focus on their experiences in Syria during the war or as refugees in countries of first asylum. I took this approach in a proactive effort to avoid concerns by the REB, I believed that had I included questions about participants’ experiences during the war, I would have been even less likely to secure approval from the REB. While there is evidence to support this strategy (Mollica 2008; Tang 2015), it became clear to me after my first round of interviews, that by studiously avoiding asking about anything to do with the war or their lives in Syria, I was reducing the cumulative life experiences of my research participants to the designation of “resettled refugee.” Because of the ongoing, pernicious, and worsening context of the war in Syria, it was and is an ongoing preoccupation of the Syrian refugee families in Canada.

While I was blithely going through my interviews asking about life in Canada, I was reproducing the same dynamics that had shaped interactions between Syrian refugees and many Canadians since their arrival—focusing on life here in Canada, treating their arrival in the place where *I* lived as the most significant event in their life, addressing immediate settlement needs, and discussing the future. It was only about 8 months into my research that I realized how out of touch my interests were with those of my research participants. When I finally, cautiously, asked Haya about my suspicions that I was missing something by not asking about Syria—she laughed and told me that in the interview we had just completed, she and the participant had spent a good part of our time together talking about the war back home, the various family members in captivity in Syria, the mounting cost of living back home, their concerns for family members, and the prospects of anti-regime forces in Syria who continued to hold out against Assad’s army (all of this took place in Arabic while I spoke to the teenage son in English). Thus, while I was careful *not* to talk about the war, this was an all-consuming, preoccupying topic of conversation for the majority of my research participants.

Ironically, by avoiding the subject of the war, Syria, or the refugee experiences of my participants, I was missing a key factor in their settlement experiences in Canada (the purported aim of my research). That is, as soon as I opened the door on this area of inquiry and started asking participants in general terms how the conflict back home was affecting their life in Canada, I came to see the profound ways in which their lives were tied to family and community well beyond the geographic confines of their current neighborhood, city, or country. The ongoing situation in Syria—which they were able to monitor through Facebook and daily conversations with family members in Syria—was the all-consuming preoccupation for my research participants, shaping how they experienced their “settlement” in Canada.

The following excerpt from my fieldnotes reflects this dynamic well:

I ask about the situation in Syria – Assad’s attacks outside of Damascus have been making the news again. H. tells me, “our heads are here but our hearts are there.” She has friends on Facebook and she talks to them sometimes; there are bombs falling and the children ask [her friend] ‘Mom, mom, save me!’ and what can she do? No one can leave Syria anymore. America, Russia, Lebanon, Iran – they are all allowing this to happen. N.’s husband A. was so upset after watching Facebook. There was a man standing in front of his car and he was so angry he wanted it to run him down. He didn’t. H. tells me, “I don’t go on Facebook because it is too upsetting. I have four kids. I need to keep going.” What about the kids, do they also talk about Syria? J. [H.’s oldest daughter] was talking to her uncle on the phone in Syria. They are in the north, near Raqqa and her uncle asked if she could come visit but it is impossible. Are they safe? No. The bombing isn’t there but Assad is trying to recruit fighters because he doesn’t have anyone left to fight for him. They are all dead, also Daesh, also [another Islamist group in the area]. . .so his kids are not safe. Why does the world let this happen? H. asks me, because we are Muslim? I shrug – I don’t know what to say. H.’s husband was sending $400/month to his mother in Syria. They fight about this. Now he sends ‘his money’ and she keeps hers. What if the [Canadian] government cuts them off or takes away the child benefit? (Fieldnote excerpt, 111417)

Here again, Lê Espiritu’s comment about the “problem of the refugee” located within the body and mind of the refugee and not within the brutal geopolitical conditions that create their displacement is relevant. By attempting to sidestep the war and its ongoing impacts, I was falling into the trap of what Wimmer and Glick Schiller (2003) have referred to as methodological nationalism—choosing to ignore the way refugee life is untethered geographically from the scales of analysis with which researchers are consumed—the nation-state, the city, the neighborhood. Speaking about the war meant acknowledging the way the war continued to shape the present and every day, rather than treating it as a past event, a thing from *before*. Indeed, there was harm in not speaking about the war and its ongoing effects, the harm of reducing specific individuals, histories, and experiences to the generalized identity of “resettled refugee.”

## Conclusion

The public preoccupation with refugee deaths. . . precludes thoughtful discussion about refugee life, not only in terms of their livelihood, which once again emphasizes the refugees’ neediness, but even more so in terms of their lived lives – how they have created their worlds and made meaning for themselves? (Lê Espiritu and Duong 2018, 597)

Ethnography is an important tool to respond to the call from critical refugee scholars to explore “how refugees have created their worlds and made meaning for themselves” (Lê Espiritu and Duong 2018). This emphasis on particularity and individual experience informed my own belief in the value of ethnography for exploring the lives of resettled refugee women (re)building their lives in Canada.

Despite the promise of ethnography for generating critical insights on the meaning-making processes of refugee families, REBs continue to police access to these purportedly “vulnerable” communities. While great care and ethical consideration must be part of all research encounters, in this case, the concerns of the REB were misaligned with the everyday realities (and vulnerabilities!) facing resettled refugee families. By suggesting that Syrian refugees are “highly vulnerable” and therefore a “high risk” research proposition, the REB reinforced a problematic idea of what it means to be a displaced person. Certainly, these families faced profound challenges, and I would argue, were often *made* vulnerable by Canadian systems, agencies, and organizations, yet they were not inherently vulnerable.

This reflects a broader challenge of the research ethics system which has uncritically adopted a narrow and fixed definition of what it means to be vulnerable or at-risk (van den Hoonaard 2018). It also reflects a problematic liberal narrative about the need to protect or save refugee populations (because, it is implied, they cannot save themselves). This narrative centers on the passivity and vulnerability of refugees and ignores their capacity to make decisions, author their lives, and exercise choice. Rather than subscribing to an uncritical view of refugees as always already vulnerable, ethnographers have a role to play in carefully drawing attention to the ways refugees make meaning for themselves in the context of displacement and resettlement.

## Notes

1 This article aims at unpacking the figure of the refugee as constructed by the REB and as such focuses less on the figure of the non-refugee researcher. Below I seek to situate myself as a researcher in relation to this project. Unfortunately, fully unpacking the role of the non-refugee researcher as constructed by the REB, while an important project in its own right, is simply beyond the scope of this article.

2 Private refugee sponsorship is a refugee resettlement program unique to Canada. It provides a way for groups of private citizens to come together to sponsor refugee families to come to Canada. 35% of Syrian refugees entering through the SRRI were privately sponsored (IRCC 2019).

3 Resettled refugees enter Canada through one of three pathways: As Government Assisted Refugees (GARs), Privately Sponsored Refugees (PSRs), or Blended Visa Office Referred Refugees (BVORs). Regardless of pathway, resettled refugees receive 1 year of financial support either from the government (GARs) or from their sponsorship group (PSRs), BVORs receive their first year of funding from a combination of public and private funds. This level of support is roughly comparable with Provincial Social Assistance rates.

4 Often the woman’s husband would be there when Haya and I arrived, we would be introduced and he would put on his jacket and leave. Other times, a husband would arrive back at the house toward the end of the interview, he would come in and say hello, and then disappear upstairs. I asked Haya about the disappearing men a few weeks into my research and she explained that it was customary for women to visit together without husbands. Had Haya’s husband or my husband accompanied us, the men would have stayed for the visit. This was confirmed when, on one occasion, the husband stayed for the duration of my visit. When Haya’s husband, Akram, came to pick us up, he made fun of the husband who had stayed for the duration of my visit, teasing “Did you stay and talk gossip with the women the whole time?” It is also possible that had I come alone, without Haya that the men may have stayed to participate. Haya’s presence signaled that this was a friendly visit between neighbors.

5 “Literacy, English and Academic Development” (LEAD) is a program for language learners in Calgary’s public school system—most Syrian children were enrolled in LEAD programs when they arrived in Canada.

6 LINC—Language Instruction for Newcomers to Canada’s language training program for immigrants and refugees to Canada

7 The decision to avoid questions related to participants’ pre-arrival experiences was a proactive attempt to stave off REB concerns over potentially “retraumatizing” refugees. This is discussed further in the final section of the article.

8 In Canada, university ethics boards are referred to as REBs in the United States, and other contexts they are referred to as “Internal Review Boards” (IRBs). The literature, therefore, refers sometimes to IRBs or REBs—for this article I use REB as it is the term used in Canada where this research took place.

9 It is not unusual, of course, for Ethics Boards to ask about issues related to child protection: Researchers are required to report child abuse in the event that they observe it. However, the specific way this question was asked by the Board positions immigrant/refugee families as potentially more deviant than non-immigrant families.
